# Alcohol in the Aging Brain – The Interplay Between Alcohol Consumption, Cognitive Decline and the Cardiovascular System

**DOI:** 10.3389/fnins.2019.00713

**Published:** 2019-07-05

**Authors:** Melinda Alicia Mende

**Affiliations:** Department of Cognitive Sciences, University of Potsdam, Potsdam, Germany

**Keywords:** cognitive decline, neuroplasticity, AUD, alcohol consumption, aging brain

## Abstract

As our society grows older new challenges for medicine and healthcare emerge. Age-related changes of the body have been observed in essential body functions, particularly in the loco-motor system, in the cardiovascular system and in cognitive functions concerning both brain plasticity and changes in behavior. Nutrition and lifestyle, such as nicotine intake and chronic alcohol consumption, also contribute to biological changes in the brain. This review addresses the effect of alcohol consumption on cognitive decline, changes in brain plasticity in the aging brain and on cardiovascular health in aging. Thus, studies on the interplay of chronic alcohol intake and either cognitive decline or cognitive preservation are outlined. Because of the inconsistency in the literature of whether alcohol consumption preserves cognitive functions in the aging brain or whether it accelerates cognitive decline, it is crucial to consider individual contributing factors such as culture, health and lifestyle in future studies.

## Introduction

During the lifespan, the human body and brain grow, develop and change. In early childhood, the body grows and cognitive functions develop (Dobbing and Sands, 1973), whereas in late adolescence, the human body and cognition begin to change and decline (Raz et al., 2005). Age-related changes have been observed in essential body functions, for instance the loco-motor system, behavioral speed (Spirduso et al., 1995) and the cardiovascular system (Bender et al., 2013) as well as in cognitive processes (Raz and Rodrigue, 2006; Salthouse, 2010; Barrett and Satpute, 2013; Kennedy and Raz, 2015).

Crucially, human life expectancy has exponentially increase over the last decades. Moreover, the age in which people retire, increases as well (Galasso, 2008) forcing society to face new challenges in healthcare and acknowledge the need for a better understanding of the physical changes in late adolescence in order to develop interventions counteracting the bodily and mental decline caused by aging.

Chronic alcohol consumption is associated with a range of negative effects on the body in all age groups, for instance liver and pancreas diseases, hepatic encephalopathy, stroke, and seizures (Zahr, 2014). Regular and extensive consumption of alcohol causes white matter loss, a reduction of brain volume and neuronal loss (Mann et al., 2001). In a survey in the year 2010, the National Institutes of Health in the United States found that 28% of adults consume alcohol at such a high level that they are exposed to the risk for the development of alcoholism (Costin and Miles, 2014). In general, addiction is characterized by use of the drug in spite of objectively observable negative effects on social and emotional functionality and well-being. Drug-seeking behavior is continued by addicts regardless of legal consequences (Konova et al., 2013). Alcoholism reaches from occasional binge drinking to a fully developed alcohol use disorder (AUD). Although, AUD is not perfectly defined, the period of time and the amount of alcohol intake play an important role. Moreover, AUD is characterized by the occurrence of life problems caused by the high consumption of alcohol (Costin and Miles, 2014).

Crucially, the amount of consumed alcohol increased in older adults of the present generation compared to the previous generation (Moussa et al., 2015). On these grounds, the current review addresses general effects of negative brain changes and cognitive decline in the aging brain and the interplay with chronic alcohol consumption.

## Changes in Cognition and Brain Plasticity in the Aging Brain

### Changes in Cognition in the Aging Brain

Some cognitive functions change and/or decline in aging. Nevertheless, cognitive processes are difficult to be categorized, for there are several different cognitive abilities, which contribute to one’s intelligence. In the middle of last century, cognitive processes were broadly categorized into so-called *fluid abilities* (also called *fluid intelligence*) on the one hand which are crucial to find solutions for new problems by using logic and finding patterns and which decline in aging. On the other hand, *crystallized abilities* (also called *crystallized intelligence*) is the use of previously explicitly learned knowledge and is less flexible than fluid intelligence. These abilities are preserved in during the lifespan (see Cattell, 1943). More recent research has found that this distinction does not hold for all cognitive functions in aging, for instance memory, which is highly sensitive to age, even if it has been classified to be one of the crystallized functions (Buckner and Louis, 2004; Salthouse, 2010).

Yet, cognitive functions cannot be mapped to anatomical brain structures in a one-to-one relationship (Salthouse, 2010), for some cognitive processes have been detected in activation patterns that are formed by several brain areas rather than in one specific area of the brain (Barrett and Satpute, 2013). Moreover, some functional cognitive brain processes improve in aging, for instance vocabulary knowledge (Salthouse, 2010), or are generally preserved, depending on their consolidation over the lifespan (Lövdén et al., 2010). Whereas other processes, for example processing speed, rapidly decline (Salthouse, 2010).

There are two main forms of cognitive decline in aging: dementia and mild cognitive impairment (Panza et al., 2012). The latter, which is named *predementia syndrome*, manifests itself in *nondemented* decline of cognitive functions, especially in memory impairment. Nondemented means that the observed syndrome does not follow any known pattern of medical diseases, but it can turn into a form of dementia (Panza et al., 2009).

Therefore, models have been established, which aim to explain changes in brain activation as a result of changes in cognitive processes in the aging brain. Table 1 shows a summary of selected models that explain cognitive decline.

**TABLE 1:** Models on Processes of Cognitive Decline in Healthy Aging.

	**Model**	**Brain Activation Patterns**	**Modified by Task Difficulty**	**Process**	**Mechanism**
A	HAROLD (Cabeza, 2002)	↑ Hemispheric lateralization	No	Reordering of neurocognitive networks	Compensation, dedifferentiation
B	PASA (Davis et al., 2008)	↓ Occipital activation ↑ Frontal activation	No	Shift in activation	Compensation
C	CRUNCH (Reuter-Lorenz and Cappell, 2008)	↑ Frontal activation ↑ Bilateral activation (in low-level cognitive tasks only)	Yes	Consumption of more resources in low-level tasks	Compensation
D	STAC (Park and Reuter-Lorenz, 2009)	↑ Frontal activation (↑ Parietal, medio-temporal, occipital activation)	No	Scaffolding of networks	Compensation

*(A) The *HAROLD model*, “hemispheric asymmetry reduction in older adults” (Cabeza, 2002): Accordingly, the reduction of lateralization may reflect a reordering of the neurocognitive networks on the anatomical level. In addition, it is assumed that there is both a compensatory process in the brain and a dedifferentiation process that cause more bilateral activation. (B) The *PASA model*, “posterior-anterior shift in aging” (Davis et al., 2008). Accordingly, there is a decrease of occipital activation in aging and increased activation in frontal regions, which is not caused by task-difficulty. Moreover, the increase in frontal activation positively correlates with performance so that it is assumed that the shift compensation mechanism, which is in line with the findings of Cabeza (2002). (C) The *CRUNCH model*, “compensation-related utilization of neural circuits hypothesis” (Reuter-Lorenz and Cappell, 2008), maps greater activation in older adults to higher task difficulty. This means that in tasks with low demand of cognitive resources, older adults show significantly more activation in frontal regions in both hemispheres than younger adults. Although, as the cognitive demand increases in tasks, younger adults show more recruitment of the frontal areas than older adults. Therefore, activation is task- dependent in this view, because only in low-demanding tasks, older adults are able to recruit more brain resources as a compensatory mechanism, whereas in tasks with a higher demand, older adults cannot recruit additional areas, since all resources are already involved. (D) The *STAC model*, “scaffolding Theory of Aging and Cognition” (Park and Reuter-Lorenz, 2009), assumes that new networks are recruited by scaffolding old networks to preserve a higher level of cognition despite anatomical changes. These networks can be observed primarily in over-activation in the frontal region, but also in the parietal cortex, medio-temporal cortex and occipital cortex. Even though these networks perform not as efficiently as cognitive networks in younger adults, the model provides an explanation for the preservation of comparatively high cognitive abilities in older adults.*

According to these models, there are cognitive mechanisms for compensating anatomical changes and reduced function of cognitive networks in the aging bran. Yet, it is not entirely clear whether more activation automatically leads to better performance in all levels of task difficulty.

### Changes in Brain Plasticity in the Aging Brain

In the last decades, numerous brain imaging studies have been conducted, particularly by using functional magnetic resonance imaging, to investigate changes in activation patterns in cognitive tasks in healthy and in clinical aging (Reuter-Lorenz and Park, 2010). This methodological development widened the focus of studies on aging from psychological assessment to both a psychology and neuroscience of aging (Cabeza, 2002; Cabeza et al., 2003).

Age-related changes in cognition are caused by biological changes in the brain. Despite inter-individual differences, environmental and genetic factors, general global changes have been detected in the aging brain, in particular age-related decline in functional brain properties, i.e., cellular, molecular and also gross anatomical structures. Cross-sectional studies have revealed that the volume of prefrontal regions, the cerebellum, hippocampus and neo-striatum in aging (Kennedy and Raz, 2015). These anatomical changes in the aging brain impact the activation patterns involved in cognition. For instance, changes in white matter, atrophy of brain tissue and neurotransmitter depletion preferably occur in the fronto-striatal systems and lead to impairment of the executive functions and the memory system in advanced aging (Buckner and Louis, 2004). Nevertheless, positive changes in brain plasticity, which have been observed in younger adults, might serve as a mechanism of compensation also in the aging brain (Reuter-Lorenz and Park, 2010). Enhancing these mechanisms by treatment and training of neurocognitive functions thus is a promising method to obviate cognitive decline (Hertzog et al., 2009).

## Influencing Factors on Changes in the Aging Brain

Several factors and interpersonal differences influence on brain plasticity in aging, for instance fitness (Kramer et al., 2006), health (Cesari et al., 2003), gender (Stampfer et al., 2005; Zanjani et al., 2013) and predisposition to diseases (Lipinski et al., 2010). Nutrition and lifestyle also influence biological changes in the aging brain, such as nicotine intake and chronic alcohol consumption (Beach et al., 2015). Studies on the interplay of chronic alcohol intake and changes in cognition and brain plasticity have revealed mixed results on whether it accelerates cognitive decline or preserves cognitive functions in the aging brain.

### Alcohol Consumption as a Factor of Cognitive Decline and Changes in Brain Plasticity

#### General Effects of Chronic Alcohol Abuse on Brain Plasticity

Chronic alcohol abuse is associated with changes in behavior, cognitive decline and changes in neuronal structure caused by complex neuroadaptations in the brain (Bjork and Gilman, 2014). In general, chronic alcohol consumption leads to degeneration of the spinal cord and the peripheral nervous system as well as malnutrition of brain cells due to changes in metabolism and lack of folate and thiamine (Vitamin B; Koike, 2019).

Alcohol abuse also severely affects the dopaminergic system, as repeated intake of alcohol increases the tolerance and suppresses to level of excitement, so that increasingly higher doses are consumed by addicts to stimulate their reward-system (for a review, see Burnett et al., 2016).

In addition, pharmacological tolerance and intensifying the dose of intake can lead to neuroinflammation and neural death (reviewed in Alfonso-Loeches and Guerri, 2011). The first studies that were conducted with tomographic scanning showed *in vivo* evidence for changes in brain plasticity in heavy drinkers (Jernigan et al., 1982; Pfefferbaum et al., 1988). Shortly after, also MRI was used to assess these changes (Pfefferbaum et al., 1992). Alcoholics and heavy drinkers have been shown to possess enlarged ventricles, whereas their overall brain volume, especially gray matter, is reduced (Kubota et al., 2001). Moreover, loss of gray matter positively correlates with years of alcohol abuse (Fein et al., 2002). In total, chronic alcohol abuse has also been found to accelerate aging-related effects (Giorgio et al., 2010). It is even possible to identify alcoholics and controls looking at MRI scans of their executive control networks and reward networks (Zhu et al., 2018). For a summary, see Table 2.

**TABLE 2:** Effects of Mild, Moderate and Heavy Drinking on Brain Plasticity, the Aging Brain and the Cardiovascular System.

**Imaging Method**	**Enlargement of** (**↑**) **/ Decrease of** (**↓**)	**Correlation with**
**Increase and Decrease in Brain Structures Related to Chronic Alcohol Consumption in Alcoholics Compared to Controls**		
CT (Jernigan et al., 1982)	↑ CSF volume	Years of consumption
CT (Pfefferbaum et al., 1988)	↑ Sulcal volume ↑ Ventricular volume	Age (↑ ventricular volume)
MRI (Pfefferbaum et al., 1992)	↑ CSF ↓ Volume of gray and white matter	Age (↓ volume of gray and white matter)
MRI (Fein et al., 2006)	↓ Cortical gray mater volume	Age and duration of abuse
MRI (Zhu et al., 2018)	↑ Changes of structural networks (executive control, reward)	–
MRI (Zahr et al., 2019)	↓ Hippocampal volume	Age

**Level of Consumption**	**Cognitive Decline** (**↓**) **/ Cognitive Preservation** (**=**) **/ Improvement of** (**↑**)	**Contributing Factors**

**Effects of Chronic Alcohol Consumption on the Aging Brain**		
Heavy (Kim et al., 2012; Ridley et al., 2013)	↓: Alcohol-related dementia (ARD)	
Heavy (Kim et al., 2012; Carrilho et al., 2013)	↓: Marchiafava-Bignami disease (MBD)	
Mild to moderate (Moussa et al., 2015)	=: Working memory, attention	
Mild to moderate (Bond et al., 2001; Spencer and Hutchison, 1999)	↑: Neuroprotective effect	
Mild to moderate (Kim et al., 2012; Reas et al., 2016)	↑: Prevention of cognitive decline and dementia	
Moderate (Panza et al., 2012)	↑: Prevention of cognitive decline and dementia	If no predisposition for Alzheimer’s disease; highest in consumption of red-wine

**Level of Consumption**	**Decline of** (**↓**) **/ Preservation of** (**=**) **/ Increase of** (**↑**)	**Contributing Factors**

**Effects of Chronic Alcohol Consumption on the Cardiovascular System**		
Heavy (Mukamal et al., 2003)	↑ Risk of cardiovascular disease	
Heavy (Kim et al., 2012; DiNicolantonio et al., 2018)	↑ Damage of cardiovascular system	Malnutrition, lack of thiamine
Mild (Kim et al., 2012)	↓ Damage of cardiovascular system	
Mild (Kim et al., 2012)	↓ Risk of myocardial infarction	Most prominent in consumption of wine
Mild (Chiva-Blanch et al., 2013)	↓ Risk of myocardial infarction	Most prominent in consumption of red wine; associated with high concentration of prophenols

#### In the Aging Brain

Research has shown that chronic, heavy alcohol consumption leads to alcohol-related dementia (ARD) which is more severe than general cognitive decline in aging (Kim et al., 2012; Ridley et al., 2013; Sabia et al., 2014). ARD is a form of dementia with symptoms that are caused by alcohol intoxication and that are observable continuously, even after the alcohol intoxication has ended. The symptoms of ARD are cognitive deficit, impairment of professional life and negative impact on social relationships, similar to the symptoms of AUD. Furthermore, chronic heavy alcohol consumption leads to degeneration and demyelination of the corpus callosum in many cases, which is associated with symptoms of the Marchiafava-Bignami disease (MBD; Kim et al., 2012). The observed symptoms of MBD start with coma and contain impairments of memory function and attention, changes of the personality and malfunction of tasks which require inter-hemispheric connection and it is caused by malnutrition, particularly the lack of vitamin B, and intoxication (Carrilho et al., 2013).

### Preservative Function of Alcohol Consumption in the Aging Brain

Chronic alcohol consumption does not automatically lead to regular binge drinking or regular heavy consumption of alcohol, since besides the regularity of the consumption, the amount of alcohol intake affects the severity of cognitive damage. Many alcohol consumers consume it on a regular basis, but on a low to moderate level. For instance, Moussa et al. (2015) found no observable cognitive decline in working memory and attention of older adults, who are regularly drinkers on a low to moderate level.

Interestingly, other studies revealed that moderate alcohol consumption even provides a protection against cognitive decline in aging. Accordingly, mild to moderate consumption of alcohol is associated with a neuroprotective effect (Spencer and Hutchison, 1999; Bond et al., 2001) and therefore prevents general cognitive decline and dementia (Kim et al., 2012; Reas et al., 2016). Panza et al. (2012) identified that the preservative effects of alcohol are highest in moderate red-wine consumption and when there is no genetic predisposition for Alzheimer’s Disease in the consumers. However as of now, it is not well understood which amount and regularity of alcohol intake, which genetic predispositions to diseases and which type of alcoholic beverage protects in the best manner. In addition, it is challenging to combine research findings, because methods and standards of definitions vary (Kim et al., 2012). Furthermore, influencing factors on health and cognition have not been equally assessed in previous studies and true abstainers were not distinguished from former drinkers. Therefore, Reas et al. (2016) suggested that in future research, drinking patterns, study populations and other co-varying factors should be assessed more carefully to ascertain that better cognitive performance in mild-drinkers is not accidentally caused by confounding co-variables.

### The Interplay of the Cardio -Vascular System, Cognitive Abilities, and Chronic Alcohol Consumption

Studies have shown that cardiovascular risk affects cognitive performance (Bender et al., 2013), that chronic alcohol consumption affects the cardiovascular system and that higher alcohol consumption increases the risk for cardiovascular disease with decline of the fibrinolytic potential (Mukamal et al., 2003).

Research on the interplay between cardiovascular health and cognitive decline in aging has suggested that improvements in cardiovascular health may lead to better neurocognitive functioning possibly by positively influencing brain plasticity. In particular, fluid intelligence and executive functions are assumed to be enhanced and preserved the more physically active patients are and the stronger their cardiovascular system is (Hertzog et al., 2009; Reuter-Lorenz and Park, 2010), as people who are physically more active have a general lower risk for physical diseases (Warburton et al., 2006). This is related to the positive relationship of cardiorespiratory function and cognitive abilities. In accordance, decline in pulmonary function is associated with impaired memory and attention (Ortapamuk and Aldoken, 2006). The vitamin thiamine has been found to be a key substance in this issue, since lack of thiamine is caused by chronic alcohol abuse and leads to damages of the cardiovascular system (reviewed in DiNicolantonio et al., 2018).

Similar to the finding on the effects of chronic alcohol consumption on cognition, excessive chronic consumption is associated with damage of the cardiovascular system, whereas low consumption affects the body in a cardio-protective way (Kim et al., 2012).

Another study assessed hemostatic factors, i.e., the level of fibrinogen and plasma viscosity, in a cross-sectional analysis. The authors found that low level alcohol consumption reduces the risk of myocardial infarction and cardiovascular disease, reflected by a lower level of cardiovascular health inducing factors. A separate analysis regarding the intake of different alcoholic beverages (beer, wine and liquor) showed that the cardio-protective effect was highest in wine-drinkers (Mukamal et al., 2001). It thus appears that low-to-moderate consumption of wine and beer positively affects the cardiovascular system. Especially red wine has been found to be protective because of its high concentration of prophenols (Chiva-Blanch et al., 2013). Crucially, this is the opposing effect compared to malnutrition caused by chronic abuse of alcohol. Table 2 summarizes the effects of mild, moderate and heavy drinking on brain plasticity, the aging brain and the cardiovascular system.

### Limitations and Future Research

Other factors highly influence alcohol tolerance. There are cultural, genetic predisposition and environmental context that contribute to differences in the amount of alcohol consumed between individuals.

Vulnerability to chronic alcohol consumption over the lifespan is modified by culture-specific custom and acceptance of drinking. For instance, Caucasians drinkers scored higher than Japanese drinkers in a cognitive test, whereas Japanese people had lower reaction times than Caucasians (Bond et al., 2003). Overall, so far more research has been conducted on cognitive decline and effects of alcohol consumption in Western societies than in other cultures (Park and Gutchess, 2006). Thus, more research is needed to warrant the generalizability of these results across cultures.

In addition, it is not assessed whether cultural differences in cognition are stronger in younger people (Hedden et al., 2002) or in older people (Gutchess et al., 2006). On these grounds, findings of cross-cultural studies are a promising way for gaining insights into universal biological mechanisms as well as the possibilities to influence aspects of cognitive abilities in aging (Park and Gutchess, 2006; Reuter-Lorenz and Park, 2010).

## Conclusion

In this review, the effects of alcohol on cognitive decline in older adults were addressed. First, models for general changes in cognition in the aging brain were reviewed. In the following, it was shown that depending on the amount and frequency of consumed alcohol, the results in cognitive tests decline or improve. Whereas in heavy drinkers cognitive decline is observed, mild-to-moderate drinking might contribute a better results in cognitive tests. Similarly, cardiovascular health might be preserved by chronic intake of low amounts of alcohol, whereas high intake damages the cardiovascular system.

Future studies are needed to investigate both cognitive performance and anatomical structures with cardiovascular factors, lifestyle factors and health as co-variables.

In addition, general mechanisms of alcohol abuse lead to changes of neuroplasticity and neural circuits. The interplay between metabolism and nutrition of brain cells, loss of structures and strength of neural circuits caused by chronic alcohol intoxication provides a complicated system of interdependences. To address all these factors, future studies will benefit from machine-learning algorithms combined with brain-imaging technologies.

## Author Contributions

The author confirms being the sole contributor of this work and has approved it for publication.

## Conflict of Interest Statement

The authors declare that the research was conducted in the absence of any commercial or financial relationships that could be construed as a potential conflict of interest.
